# Associations between adverse childhood experiences and medical students’ interest in careers: a single-setting study

**DOI:** 10.3389/fpsyt.2025.1483871

**Published:** 2025-03-04

**Authors:** Phillip Yang, Barbara Robles-Ramamurthy, Kristen A. Plastino

**Affiliations:** ^1^ Joe R. and Teresa Lozano Long School of Medicine, UT Health San Antonio, San Antonio, TX, United States; ^2^ Department of Psychiatry and Behavioral Sciences, Joe R. and Teresa Lozano Long School of Medicine, UT Health San Antonio, San Antonio, TX, United States; ^3^ Department of Obstetrics and Gynecology, Joe R. and Teresa Lozano Long School of Medicine, UT Health San Antonio, San Antonio, TX, United States

**Keywords:** adverse childhood experiences, medical education, career choices, medical students, surveys and questionnaires, psychiatry, academic medicine, mental health

## Abstract

**Background:**

Adverse childhood experiences (ACEs) are pervasive across communities, including medical students and physicians. Exposure to childhood trauma influences career decisions, such as social workers and nurses. However, the impact of ACEs on medical students’ career interests remains unexplored.

**Methods:**

From August to October 2022, a survey was designed and administered to medical students at UT Health San Antonio School of Medicine (Texas, USA). Ten household-level and four community-level ACEs were assessed. Associations between ACE distribution and interest in medical specialties, academia, and primary care were analyzed by Mann-Whitney *U* test.

**Results:**

Four hundred nineteen (47.0%) of 891 total students completed the survey, of which 310 (74.3%) reported at least one ACE and 107 (25.7%) reported four or more. The most common ACE was living with a household member with mental illness (154, 36.9%). Students interested in psychiatry (*p*<.01) or academic medicine (*p*=.02) had significantly higher ACE scores than those not interested in these fields. No associations were observed between ACEs and students’ interest in primary care.

**Discussion:**

The prevalence of medical students living with a household member with mental illness was approximately double than reported in population-based studies. Childhood trauma exposure may influence medical students’ interests in psychiatry and academic medicine careers. Further research is needed to investigate how ACEs influence medical students’ career considerations. Research exploring ACEs exposure in academic physicians and psychiatrists may further illuminate the associations found in this brief report. Importantly, advancements in trauma-informed approaches to medical education are necessary to facilitate safe learning environments.

## 1 Introduction

Significant personal experiences, such as personal or familial illness, are substantial reasons that influence individuals to pursue a career in medicine (1). Adverse childhood experiences (ACEs) are potentially traumatic childhood experiences that have a graded dose-response relationship on lifetime mental health challenges, health conditions, and mortality (2). Within the literature, ACEs are most commonly categorized as 10 items within households on abuse (e.g., physical, sexual, emotional), neglect (e.g., emotional, physical), and household dysfunction (e.g., domestic violence, household substance abuse, household mental illness, incarcerated household member, parental separation/divorce) (2). Community-level ACEs (i.e., witnessing or experiencing community violence, experiencing discrimination) have been identified to expand on experiences of childhood trauma among diverse urban communities (3). ACEs are common, and even more prevalent among racial minorities. In a study of 214,157 adults across 23 states, 61.6% had at least one ACE while 15.8% had four or more (4). In a socioeconomically and racially diverse urban Philadelphia, PA community, 72.9% of 1,784 participants had at least one household-level ACE and 63.4% had at least one community-level ACE (3).

Experiences of childhood trauma may play an important role in the construction of career choices. Some individuals choose careers that rectify meaningful personal problems such as unresolved aspects of childhood trauma (5). This has been explored in helping professions, where cumulative childhood trauma influences identity formation and skill development (i.e., altruism, empathy, creativity, resilience) (6). The trauma-precipitated identity and skills are then incorporated in career decisions to re-author personal trauma narratives (7). Several helping professions have been found to have elevated rates of ACEs compared to the general population. In a national study of 1,094 nursing students, 83.7% had at least one ACE while 40.7% had four or more ACEs (8). In 5,540 licensed social workers across 13 states, 70.3% had at least one ACE while 23.6% had four or more ACEs (9). Carl Jung’s wounded healer archetype, where the provider must be wounded to heal effectively, has been applied to these professions whereby lived experiences may enhance the provider’s ability to offer compassionate care (10). However, this same empathy may leave healthcare professionals vulnerable to secondary or vicarious trauma, as repeated exposure to patients’ suffering can lead to compassion fatigue and emotional exhaustion (11). The dual burden of personal wounds and vicarious trauma underscores the importance of self-awareness and healing within the wounded healer. Research on ACEs within the medical profession, however, is limited. Several single-setting studies found comparable levels of ACEs in medical students and physicians to the general population (12–15).

Medical students are a unique population known to experience high levels of psychological distress, with many factors contributing to their vulnerability. The phenomenon of imposter syndrome is prevalent in this group, where students may feel inadequate or fear being unworthy of their role as future physicians (16). This mindset, combined with traits such as maladaptive perfectionism—characterized by rigid expectations, fear of mistakes, and setting unattainably high goals—can predispose students to anxiety, depression, and chronic fatigue (17). The competitive and often high-pressure medical school environment further exacerbates these stressors, leading to significant rates of burnout (18, 19). During the COVID-19 pandemic, learning challenges were amplified due to the transition to online education and medical students faced heightened psychological distress, particularly among female students and those with pre-existing mental health conditions (20). Notably, findings during this period revealed a nuanced picture. While anxiety and substance use increased among U.S. medical students, rates of burnout, self-harm, and suicide decreased (21). These vulnerabilities may intersect with students’ motivations for pursuing a medical career, particularly in individuals with personal experiences of suffering or trauma. ACEs, for example, can shape emotional resilience and influence career decisions, driving some toward medicine while also contributing to their susceptibility to stress.

Research has identified the importance of economic factors and lifestyle on medical students selecting specialty choices (22). Other work in career decisions has focused on medical students interested in a specific specialty (23). Careers in academic medicine can be attractive for medical students due to opportunities to advance the field of medicine and teach and train the next generation of leaders (24). However, many lose interest in academia due to unsupportive environments, financial considerations, and workload challenges (25). Similarly, in primary care careers, increased responsibilities and decreased clinical autonomy can lead to greater burnout, which contributes to the increased shortage of primary care physicians (26). Careers in medicine are broad with over 160 specialties/subspecialties that can be applied in various settings such as primary care or academia. The objective of our preliminary study was to characterize associations between ACE scores in medical students and career interests in medical specialty, primary care, and academic medicine.

## 2 Materials and methods

### 2.1 Subjects

Medical students enrolled at UT Health San Antonio School of Medicine, a public medical school in Texas, USA, were eligible to participate in this study from August to the end of October 2022. The survey was emailed once each month for a total of three times to all medical students via the medical student listserv. We advertised the survey during one lecture for the MS-1, MS-2, and MS-3 classes. The MS-4 class did not have any class-wide lectures during that period. We also posted flyers with the survey on a QR code next to medical lecture rooms. As an incentive to complete the survey, we raffled five $100 gift cards for a large chain grocery store or gas station. The investigation was determined human research exempt by our institution’s Institutional Review Board.

### 2.2 Measures

We developed the survey based on discussions with our institution’s Office of Undergraduate Medical Education and a review of the literature. Demographic variables selected include year of medical education, age, gender, and race/ethnicity. For medical specialty, participants indicated one specialty that they were currently most interested in. Participants indicated their interest in primary care and academia using a 5-point Likert scale. Definitely not or Probably not responses were scored No and Probably yes and Definitely yes were scored Yes. Primary care was described as, Primary care physicians are the initial doctors that patients approach for treatment. Primary care specialties can include family medicine, general internal medicine, general pediatrics, combined internal medicine/pediatrics (meds/peds), and general obstetrics and gynecology. Academia was described as, Academic medicine is a broad career which can include a variety of scholarly activities, such as research, teaching, administrative, and managerial roles. In the Adverse Childhood Experiences block, we utilized the original 10-question household-level adverse childhood experiences questionnaire in addition to four community-level ACEs adapted from the literature (2, 3). We used Qualtrics (Qualtrics, Provo, UT) for survey management. The survey is available upon reasonable request.

### 2.3 Statistical analysis

While the ACE score distribution was nonparametric, we descriptively reported both median (IQR) and mean (SEM) ACE scores for ease of inter-study comparison. Mann-Whitney *U* test of medians was utilized to investigate differences in ACE distribution between participants interested in medical specialties, academia, and primary care and those not interested in these fields. In line with other ACEs research, ACE scores were treated as a continuous variable, with scores of 6 or higher combined due to limited size. We used Prism 10.1.2 (GraphPad Software, San Diego, California) to analyze our data.

## 3 Results

### 3.1 Demographics 

Four hundred nineteen participants (response rate of 47.0%) of 891 total students completed the survey (**Table 1**). Two participants did not complete most ACEs questions and were excluded from this study, leaving an analytical sample size of 417 participants (46.8%). The average age was 24.4 (*SD* = 2.7) years. Most participants were preclinical (MS1 and MS2) students (135, 32.4%; 136, 32.6%, respectively), identified as women (237, 56.8%), and were White (163, 39.1%) or Asian (135, 32.4%).

**Table 1:** Demographics of medical students at a Southeast public medical school (N=417).

Age in years mean (SD)	24.4 (2.7)
Medical Student (MS) Year	n (%)
MS1	135 (32.4)
MS2	136 (32.6)
MS3	76 (18.2)
MS4	66 (15.8)
Gap Year	4 (1.0)
Gender	n (%)
Man	174 (41.7)
Woman	237 (56.8)
Non-binary/Non-conforming	6 (1.4)
Race/Ethnicity	n (%)
American Indian	3 (0.7)
Asian	135 (32.4)
Black	15 (3.6)
Hispanic	52 (12.5)
Multiracial	48 (11.5)
Native Hawaiian or Other Pacific Islander	1 (0.2)
White	163 (39.1)

SD, standard deviation.

### 3.2 ACE score distribution

The median and mean household-level ACE scores were 1 (IQR = 3) and 1.77 (SEM = 0.10), respectively (**Table 2**). The median and mean total ACE score were 2 (IQR = 4) and 2.45 (SEM = 0.12), respectively. The three most reported household-level ACEs were household mental illness (154, 36.9%), emotional abuse (108, 25.9%), and parental separation/divorce (106, 25.4%). Two hundred seventy-two (65.2%) participants reported at least one household-level ACE and 76 (18.2%) participants reported four or more. For community-level ACEs, 107 (25.7%) participants reported experiencing severe bullying, 85 (20.4%) reported experiencing racism, 61 (14.6%) experienced community/neighborhood violence, and 27 (6.5%) experienced sexism or genderism. When accounting for both household-level and community-level ACEs, 310 (74.3%) participants reported at least one ACE and 107 (25.7%) participants reported four or more.

**Table 2:** ACE distribution in medical students at a Southeast public medical school (N=417).

Household-level ACEs	n (%)
Household depressed, mentally ill, or suicidal	154 (36.9)
Emotional abuse	108 (25.9)
Parental separation/divorce	106 (25.4)
Witnessed domestic violence	85 (20.4)
Physical abuse	78 (18.7)
Emotional neglect	76 (18.2)
Household alcohol/drug abuse	66 (15.8)
Sexual abuse	35 (8.4)
Physical neglect	15 (3.6)
Household incarceration	15 (3.6)
Community-level ACEs	n (%)
Experienced being bullied	107 (25.7)
Experienced racism	85 (20.4)
Community/Neighborhood violence	61 (14.6)
Experienced sexism/genderism	27 (6.5)
Household-level ACE Score	n (%)
0 Household-level ACEs	145 (34.8)
1 Household-level ACEs	90 (21.6)
2 Household-level ACEs	70 (16.8)
3 Household-level ACEs	36 (8.6)
4 Household-level ACEs	36 (8.6)
5 Household-level ACEs	13 (3.1)
6+ Household-level ACEs	27 (6.5)
ACE Score Median (IQR)	1 (3)
ACE Score Mean (SEM)	1.77 (0.10)
Household and Community-level ACE Score	n (%)
0 Total ACEs	107 (25.7)
1 Total ACEs	88 (21.1)
2 Total ACEs	68 (16.3)
3 Total ACEs	47 (11.3)
4 Total ACEs	26 (6.2)
5 Total ACEs	31 (7.4)
6+ Total ACEs	50 (12.0)
ACE Score Median (IQR)	2 (4)
ACE Score Mean (SEM)	2.45 (0.12)

ACE, adverse childhood experience; IQR, interquartile range; SEM, standard error of the mean.

### 3.3 ACE score and career interests

**Figure 1:**
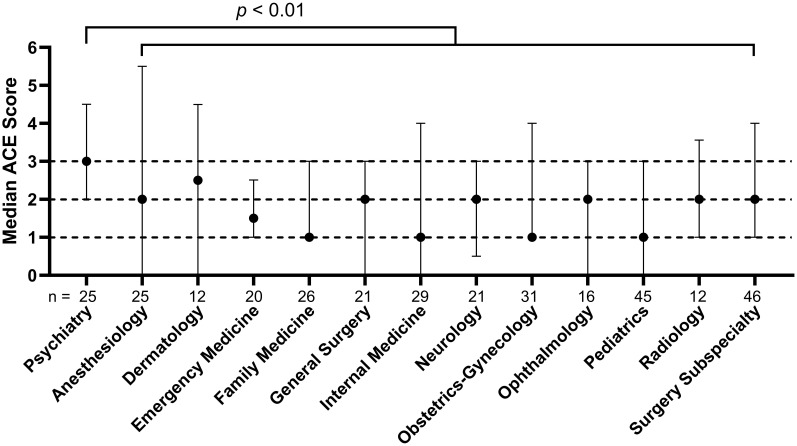
Adverse childhood experiences (ACE) score and U.S. medical students’ interest in medical specialties Dotted lines placed at median ACE score of two and three for visualization. Error bars are interquartile range. Mann-Whitney *U* test of ACE scores between students interested in psychiatry vs non-psychiatry specialties.

**Figure 2:**
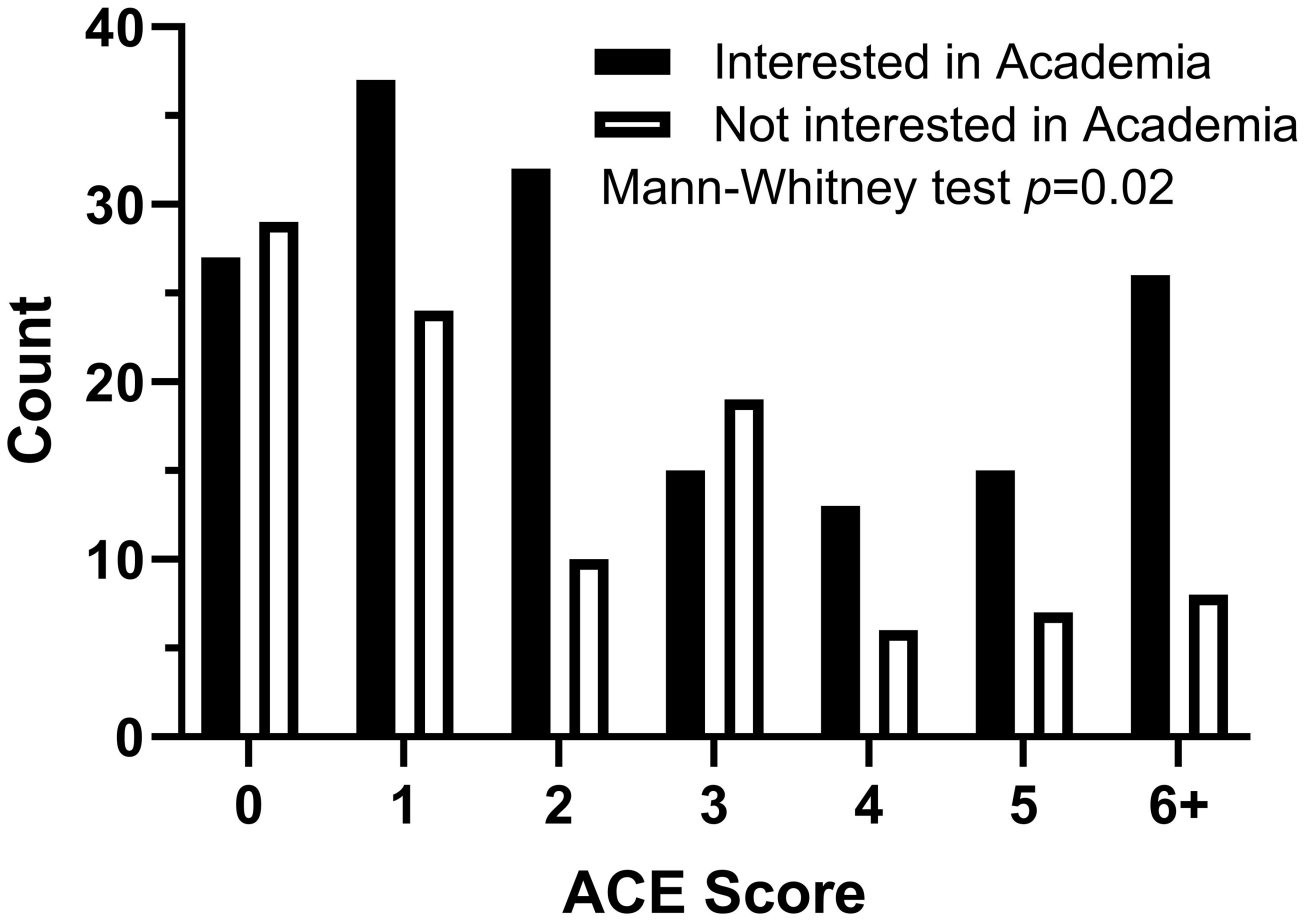
Adverse childhood experiences (ACE) score and U.S. medical students’ interest in academia Mann-Whitney *U* test of ACE score distribution between students interested in academia vs not interested in academia.

Participants indicated 37 unique medical specialties of interest. The 14 specialties with a sample size greater than 10 are displayed in **Figure 1**. The distribution of ACEs among participants interested in psychiatry was significantly greater than among participants not interested in psychiatry (Mann–Whitney *U* = 3319, n_1_ = 25, n_2_ = 392, *p* < 0.01 two-tailed). One hundred forty-one (33.8%) and 167 (40.0%) participants were interested in a career in primary care and academia, respectively. The distribution of ACEs among participants interested in academia was significantly greater than among participants not interested in academia (**Figure 2**; Mann-Whitney *U* = 7106, n_1_ = 165, n_2_ = 103, *p* = 0.02 two-tailed). There was no statistically significant difference in ACE distribution and interest in primary care.

## 4 Discussion

Similar to the other published works on ACEs in medical students, our sample has comparable ACE scores to the general population (12, 13). All three studies reported living with a household member with mental illness as the most frequently experienced ACE by medical students (29.0% - 36.9%) while large representative general population studies have reported lower frequencies between 16.5%-17.5% (2–4, 12, 13). Experience with personal or familial illness among medical students, trainees, and physicians has been reported to promote compassionate patient care and empathy for patients (1). These meaningful experiences may have also driven individuals to pursue careers in medicine. Our work is the first to report community-level ACEs in medical students and that they are common, with approximately one-quarter (25.7%) reporting frequent bullying and one-fifth reporting frequent racism (20.4%). In total, roughly three-quarters (74.6%) of our sample experienced at least one of the 14 ACEs and one-quarter (25.8%) experienced four or more.

Elevated ACEs scores have been found in various mental health professionals, including social workers, professional counselors, and psychologists (9, 27, 28). In a large international survey of final-year medical students, those with personal exposure to mental illness or a history of mental health care were more likely to choose psychiatry as their medical specialty (23). This trend has yet to be investigated in psychiatrists; however, our study shows medical students who were interested in psychiatry had higher ACE scores. Cumulative childhood trauma is associated with mental health challenges and may lead students with higher trauma burden to use their experiences to care for future patients and re-author their trauma narrative (1, 2). Forty percent of our sample was interested in a career in academic medicine and those with exposure to ACEs were more likely to be interested in academia than those without ACE exposure. Exposure to ACEs is a public health issue with physical and mental health consequences. While clinical work may treat health consequences of ACEs, medical students with ACEs exposure may be interested in academia due to its broader potential to combat factors associated with their experiences of trauma through research, teaching, and community work. In contrast, interest in primary care does not appear to have an association with ACE burden in our sample.

Experiences of childhood trauma and significant stress can result in hypervigilance and emotional reactivity, which when combined with the stress of clinical education, can place students at a higher risk of emotional stress, mental health conditions, and burnout (29). While many medical schools offer school-based mental health services, few schools provide comprehensive student insurance coverage for mental health treatment (30). Medical students also experience feelings of stigma and shame when seeking mental health services (31). Free, anonymous, and unrestricted access to mental health services is necessary to lower the barriers to seeking care. For instance, in 2020-2021, a large academic training hospital introduced an opt-out mental health appointment for resident physicians, which helped lower barriers to accessing care, encouraged greater participation in mental health services, and cultivated a supportive institutional culture around well-being (32). Innovations in trauma-informed medical education have only recently begun being designed and implemented. Brown et al. (33) described the Trauma-Informed Medical Education (TIME) framework at Harvard Medical School which comprehensively teaches students and faculty trauma-informed care clinical practices and self-care techniques. Importantly, the TIME framework emphasized applying trauma-informed care principles in the educational context. In this way, students, especially those with trauma history, are in a safe and productive environment to access trauma-informed care content and minimize the risk of re-traumatization. Furthermore, intentional effort must elevate diverse voices when implementing trauma-informed approaches to adequately capture the needs of under-represented minority individuals, who may have experienced greater childhood trauma and career-related stressors (4). This concern has been documented in the higher rates of dismissal experienced by Black medical students and those who come from lower-income households and under-resourced neighborhoods (34).

### 4.1 Limitations

Psychometric validation of the original ten-item household-level ACEs questionnaire and the community-level ACEs questionnaire are largely lacking. The ACE questionnaire was initially developed for epidemiological studies rather than a clinical screening tool, leading to less emphasis on traditional psychometric validation (35). The ten-item ACEs questionnaire has been used extensively in research and adapted by organizations like the CDC for population-representative surveys, underscoring its widespread acceptance and utility.

Our study is the largest single-setting study investigating ACEs in U.S. medical students. However, our results are limited to our institution. We need more investigations of ACEs within medical schools and academic settings to adequately understand and promote trauma-informed changes across educational curricula and institutional policy. While a significant portion of the medical student body was represented in our survey, our convenient sampling method may bias toward participants with trauma exposure. It is also important to note that our study only evaluated interest in careers and not ultimate career decisions. Career interests among medical students are dynamic, often evolving with various experiences such as clinical exposure. A qualitative study of medical students with ACE exposure could describe meaningful patterns on how trauma exposure may influence career interests and decisions. Future work could also follow medical students to assess how ACEs may influence career choices throughout medical school and how to support their health and healing processes as they learn to care for others. Finally, our cross-sectional study design prohibits causality from being inferred.

### 4.2 Next steps and conclusions

Our findings suggest that childhood trauma is a significant factor influencing medical students’ career interests. Specifically, ACEs appear to be particularly prevalent among medical students with an interest in psychiatry and academia. Future research should investigate whether this initial interest in psychiatry translates into a commitment to psychiatry as a career. Further exploration of how childhood trauma interests with other factors, such as race/ethnicity, gender, or other formative life experiences, is warranted to better understand its impact on ultimate career decisions in medical students. Academic institutions should prioritize the development and implementation of trauma-informed educational curricula and policies. Cultivating a trauma-informed culture among students, trainees, staff, and faculty could foster a more supportive environment, ultimately enhancing well-being and professional development for all.

## Data Availability

The raw data supporting the conclusions of this article will be made available by the authors, without undue reservation.
